# Cadonilimab as second-line therapy in immunotherapy-resistant squamous NSCLC: a case report and review

**DOI:** 10.3389/fimmu.2025.1627147

**Published:** 2025-07-24

**Authors:** Shuo Qiu, Mingtao Shi, Zhiying Chen, Jing Wang, Huanliang Cui, Yongchun Zhang

**Affiliations:** Department of Radiation Oncology, The Affiliated Hospital of Qingdao University, Qingdao, China

**Keywords:** immunotherapy, squamous non-small cell lung cancer, immunotherapy resistance, PD-1/CTLA-4 bispecific, case report

## Abstract

Immune checkpoint inhibitors (ICIs) have become a pivotal therapeutic option for the treatment of advanced non-small cell lung cancer (NSCLC), particularly as a standard first-line therapy. However, most patients eventually develop resistance to ICIs, and the options for second-line treatment remain limited with suboptimal efficacy. Cadonilimab, a novel bispecific antibody targeting programmed death-1 (PD-1) and cytotoxic T-lymphocyte-associated antigen 4 (CTLA-4), has demonstrated promising antitumor activity with a manageable safety profile. Nevertheless, its clinical efficacy in patients who have developed resistance to prior immunotherapy remains largely unexplored. This report presents a case of an elderly patient with early-stage NSCLC who developed resistance following first-line immunotherapy. After receiving subsequent treatment with cadonilimab, the patient achieved a partial response (PR) at the third cycle. The patient experienced substantial clinical improvement, including marked relief from chest tightness and shortness of breath, as evidenced by a reduction in modified Medical Research Council (mMRC) dyspnea grade from 3 to 1. The quality of life improved significantly, as indicated by a rise in the Karnofsky Performance Status (KPS) score from 60 to 80. Progression-free survival (PFS) was extended to 17 months, and the patient continues to derive clinical benefit. No immune-related adverse events (irAEs) affecting daily life occurred throughout the entire course of therapy. These findings suggest that cadonilimab may serve as a promising subsequent-line therapeutic option for patients with immunotherapy resistance.

## 1 Introduction

Lung cancer remains the most frequently diagnosed malignancy worldwide, accounting for approximately 12.4% of all new cancer cases and contributing to as many as 18.7% of cancer-related deaths globally (1). NSCLC, which constitutes nearly 85% of all lung cancer diagnoses, continues to pose a major public health burden (2). Although significant progress has been made in recent therapeutic approaches, the overall five-year relative survival rate for NSCLC remains disappointingly low at approximately 22%. The prognosis varies markedly by disease stage: patients diagnosed at stage I exhibit a five-year survival rate of around 65%, whereas those with stage IV disease experience a dramatic decline in survival, with rates approaching only 5% (2).

From a histological perspective, adenocarcinoma is the most prevalent subtype of NSCLC, accounting for approximately 40% of cases, whereas squamous cell carcinoma (SCC) constitutes about 20% to 30% (3). Compared with lung adenocarcinoma (LUAD), patients diagnosed with lung squamous cell carcinoma (LUSC) derive limited therapeutic benefit from targeted therapies due to the absence of well-defined actionable molecular alterations. In recent years, immune checkpoint inhibitors (ICIs) have demonstrated significant advancements in the treatment of NSCLC. Immunotherapy targeting the PD-1/PD-L1 pathway has increasingly been adopted as a first-line treatment option, either as monotherapy or as part of combination regimens. However, in clinical practice, an estimated 30% to 50% of patients either fail to respond to initial immunotherapy or develop resistance over time (4). This resistance not only accelerates disease progression but also exacerbates tumor-related symptoms such as dyspnea and pain, thereby significantly impairing patients’ quality of life and increasing caregiver burden. Overcoming this challenge remains a critical unmet need in oncology care. Given the limited efficacy associated with conventional second-line interventions, including chemotherapy, there is a pressing need to explore more effective rechallenge strategies. Cadonilimab represents the first PD-1/CTLA-4 bispecific antibody independently developed in China. By simultaneously targeting both PD-1 and CTLA-4 within a single molecular structure, cadonilimab is engineered to enhance antitumor immune responses and synergistically activate tumor-specific T-cell activity (5). Additionally, it exhibits a more favorable safety profile compared with conventional combination immunotherapies. Although emerging evidence suggests that cadonilimab demonstrates promising antitumor activity across multiple malignancies, clinical data supporting its use in patients who have developed resistance to ICIs—particularly in the context of LUSC—remain limited.

In this report, we present a case of an elderly patient diagnosed with programmed death-ligand 1 (PD-L1) high-expression squamous non-small cell lung cancer (tumor proportion score [TPS] ≥ 50%) who had previously developed resistance to PD-1 monoclonal antibody therapy. The patient achieved a PR by the fourth treatment cycle following later-line administration of cadonilimab (375 mg, 6 mg/kg), with a progression-free survival (PFS) of 17 months. Although further validation through larger clinical trials is required to confirm the efficacy of this therapeutic approach, this case may offer a meaningful reference for the management of patients who have developed resistance to ICIs.

## 2 Case description

### 2.1 Medical history

The patient was a 72-year-old male who presented to our hospital on November 20, 2020, complaining of a two-year history of chest tightness and dyspnea. He had a medical history significant for chronic obstructive pulmonary disease (COPD) for six years, hypertension for ten years—with a peak recorded blood pressure of 210/140 mmHg—and coronary atherosclerotic heart disease for five years. He had previously undergone lumbar disc surgery. For antihypertensive management, the patient had been self-administering reserpine with irregular blood pressure monitoring, maintaining his blood pressure generally within the range of 140–160/90–100 mmHg. Additionally, he had a 35-year smoking history (10 cigarettes per day) and had successfully quit smoking two years prior to presentation. Imaging studies revealed left adrenal gland thickening and a hepatic hemangioma.

### 2.2 Diagnosis

**Figure 1:**
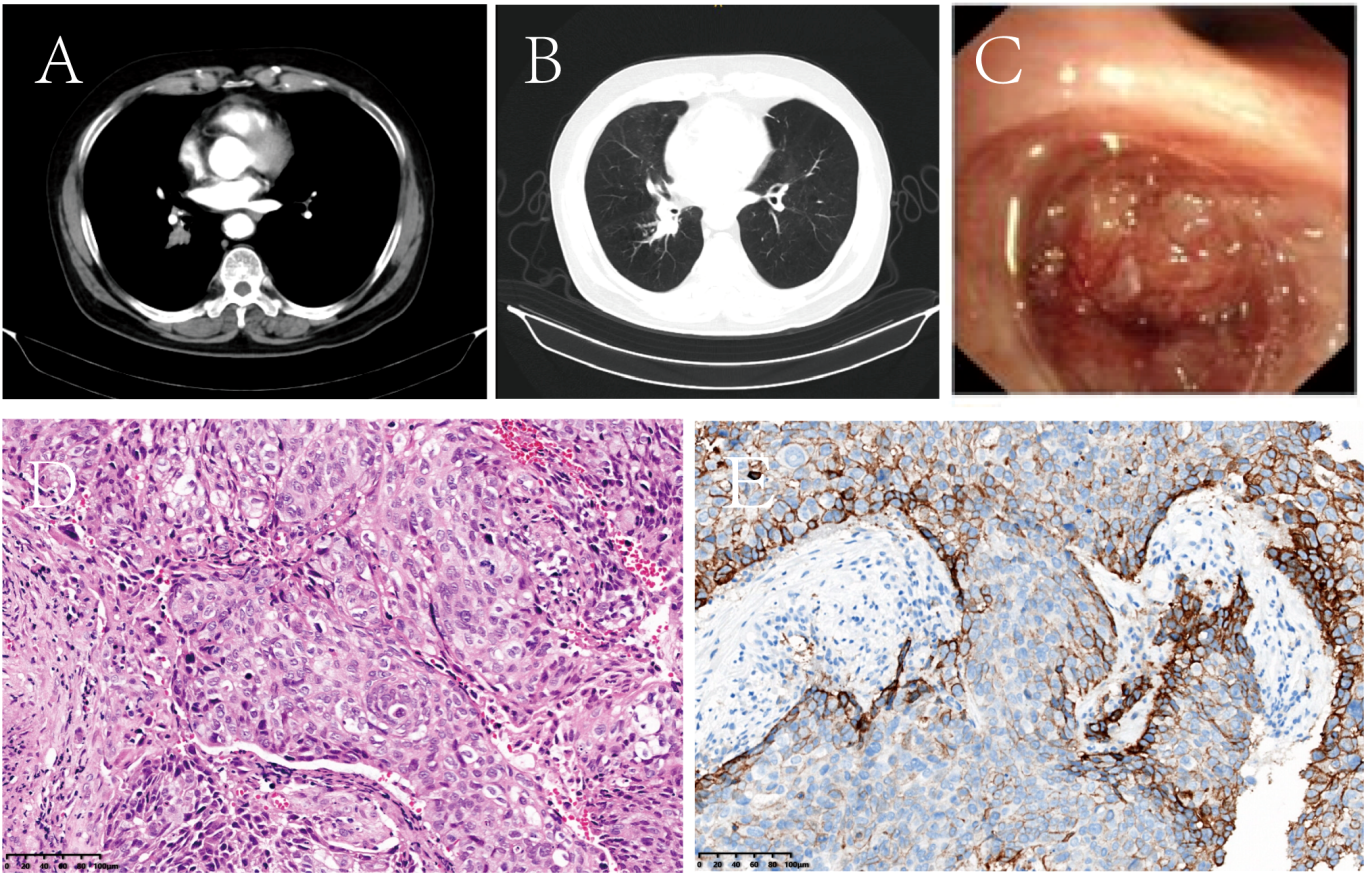
Representative baseline diagnostic assessments prior to treatment initiation. **(A, B)** Contrast-enhanced chest CT demonstrated a space-occupying lesion in the right lower hilar region, accompanied by obstructive pneumonia. **(C)** Bronchoscopic examination revealed an endobronchial mass causing luminal obstruction of the dorsal segment of the right lower lobe. **(D)** Histopathological analysis with hematoxylin and eosin (H&E) staining confirmed poorly differentiated squamous cell carcinoma (original magnification, ×100). **(E)** Immunohistochemical staining revealed PD-L1 expression with a tumor proportion score (TPS) of 55%.

On November 26, 2020, contrast-enhanced chest computed tomography (CT) identified a 19.2 mm mass-like soft tissue density in the right lower lobe hilar region, accompanied by obstructive pneumonia (**Figures 1A, B**). Subsequent bronchoscopy revealed a luminal-obstructing mass located in the dorsal segment of the right lower lobe (**Figure 1C**). Histopathological analysis of the biopsy specimen confirmed poorly differentiated squamous cell carcinoma (**Figure 1D**). A comprehensive clinical and radiological evaluation showed no evidence of distant metastasis. According to the 8th edition of the American Joint Committee on Cancer (AJCC) TNM staging system, the patient was staged as cT2N0M0. Molecular testing demonstrated negative results for both EGFR mutations and ALK gene rearrangements. Immunohistochemical staining revealed a PD-L1 tumor proportion score (TPS) of 55% (**Figure 1E**). The patient’s performance status was assessed as Eastern Cooperative Oncology Group (ECOG) grade 1.

Based on the integrated clinical findings, the final clinical diagnoses included primary lung cancer (SCC), COPD, stage III hypertension (high-risk category), status post lumbar discectomy, nodular adrenal gland thickening, and hepatic hemangioma.

### 2.3 Treatment timeline and radiographic response

#### 2.3.1 First-line immunotherapy phase

**Figure 2:**
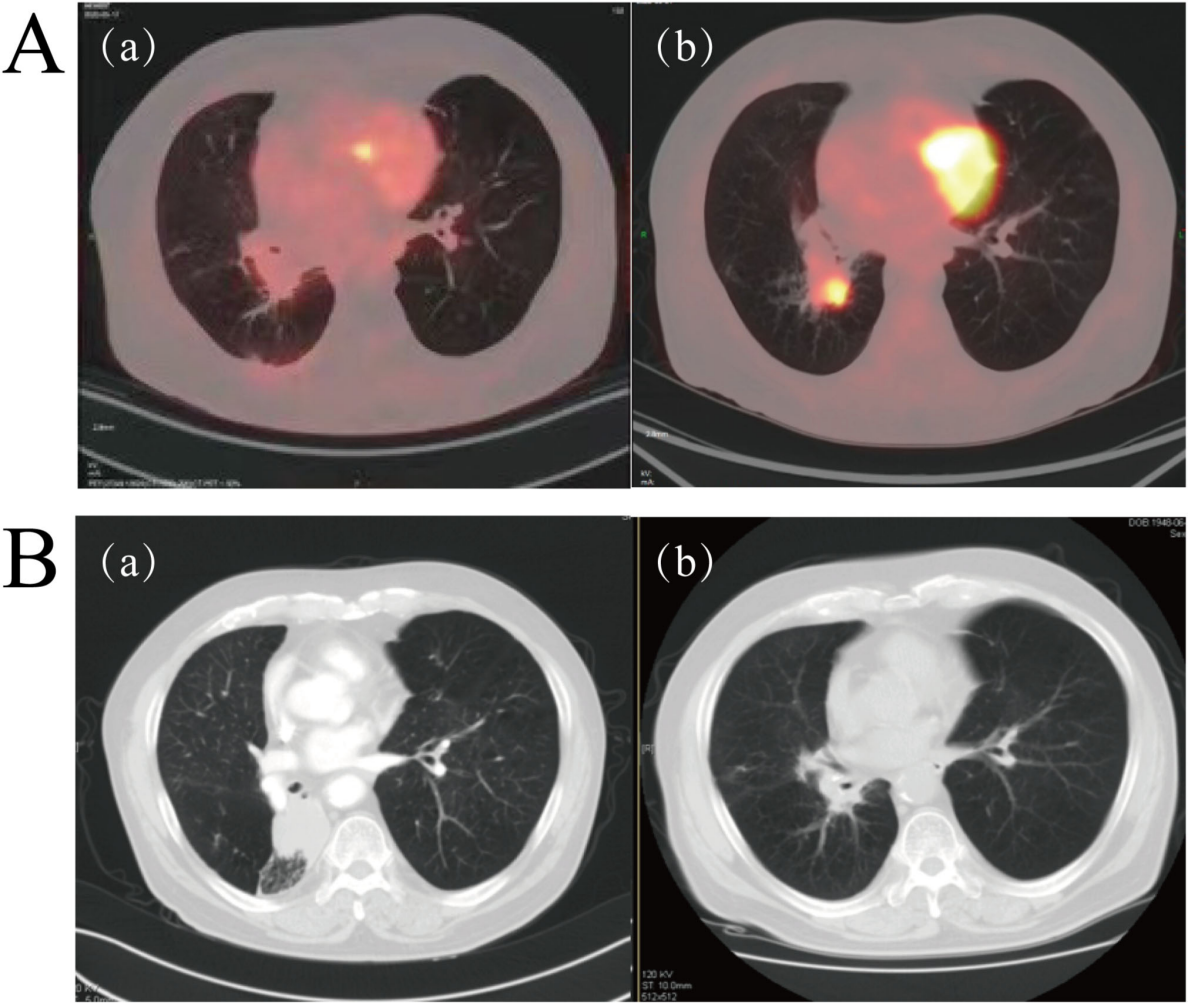
**(A)** Representative PET/CT imaging during pembrolizumab therapy. **(a)** PET/CT performed on April 7, 2021, showed a marked reduction in the size of the right lower lobe lesion compared to baseline (**Figure 1A**). (b) PET/CT conducted on March 31, 2022, demonstrated significant progression of the lesion in the right lower lobe relative to image **(A, B)** Serial contrast-enhanced chest CT scans before and after radiotherapy. (a) CT scan obtained on November 21, 2022, prior to radiotherapy. (b) CT scan performed on February 20, 2023, approximately six weeks post-radiotherapy completion, indicating complete response (CR).

Following a multidisciplinary team (MDT) discussion involving specialists from thoracic surgery, radiology, oncology, and pathology departments, it was concluded that surgical intervention would pose a high risk to the patient due to advanced age, poorly controlled hypertension, and underlying pulmonary disease. Moreover, the patient’s family explicitly declined both surgical resection and chemoradiotherapy. Consequently, the patient initiated pembrolizumab as first-line therapy on December 28, 2020. Encouragingly, after completing four cycles of treatment, follow-up PET/CT imaging demonstrated a reduction in the primary lesion to 9.0 × 14 mm (**Figure 2A**, region a), fulfilling the RECIST version 1.1 criteria for PR. The patient reported substantial relief from symptoms including chest tightness and dyspnea, along with notable improvements in general condition and daily functional capacity. Pembrolizumab therapy was therefore continued. On March 31, 2022, subsequent PET/CT imaging revealed disease progression (PD), characterized by an increase in the size of the right lower lobe hilar mass to 27 × 34 mm and the emergence of a new hypermetabolic lymph node in the left hilar region (SUVmax 8.7) (**Figure 2A**, region b). The patient achieved a PFS of 16 months with first-line immunotherapy.

#### 2.3.2 Local salvage therapy phase

Following disease progression, local radiotherapy was recommended. However, the patient’s family initially declined and opted for traditional Chinese medicine instead. On November 21, 2022, contrast-enhanced CT imaging revealed further tumor enlargement, with the lesion measuring 48 mm × 38 mm (**Figure 2A**, region a), confirming progressive disease. Subsequently, on November 30, 2022, the patient underwent local radiotherapy targeting the lesion in the right lower lobe, receiving a total radiation dose of 60 Gy delivered in 30 fractions. A follow-up CT scan conducted one and a half months post-radiotherapy demonstrated a complete radiological response (CR) (**Figure 2B**, region b). Throughout the treatment course, the patient tolerated the therapy well without experiencing significant adverse events such as radiation pneumonitis. Unfortunately, the patient did not adhere to scheduled follow-up visits after completing radiotherapy, underscoring the importance of improving post-treatment health management strategies and enhancing patient education.

#### 2.3.3 Second-line immunotherapy phase

**Figure 3:**
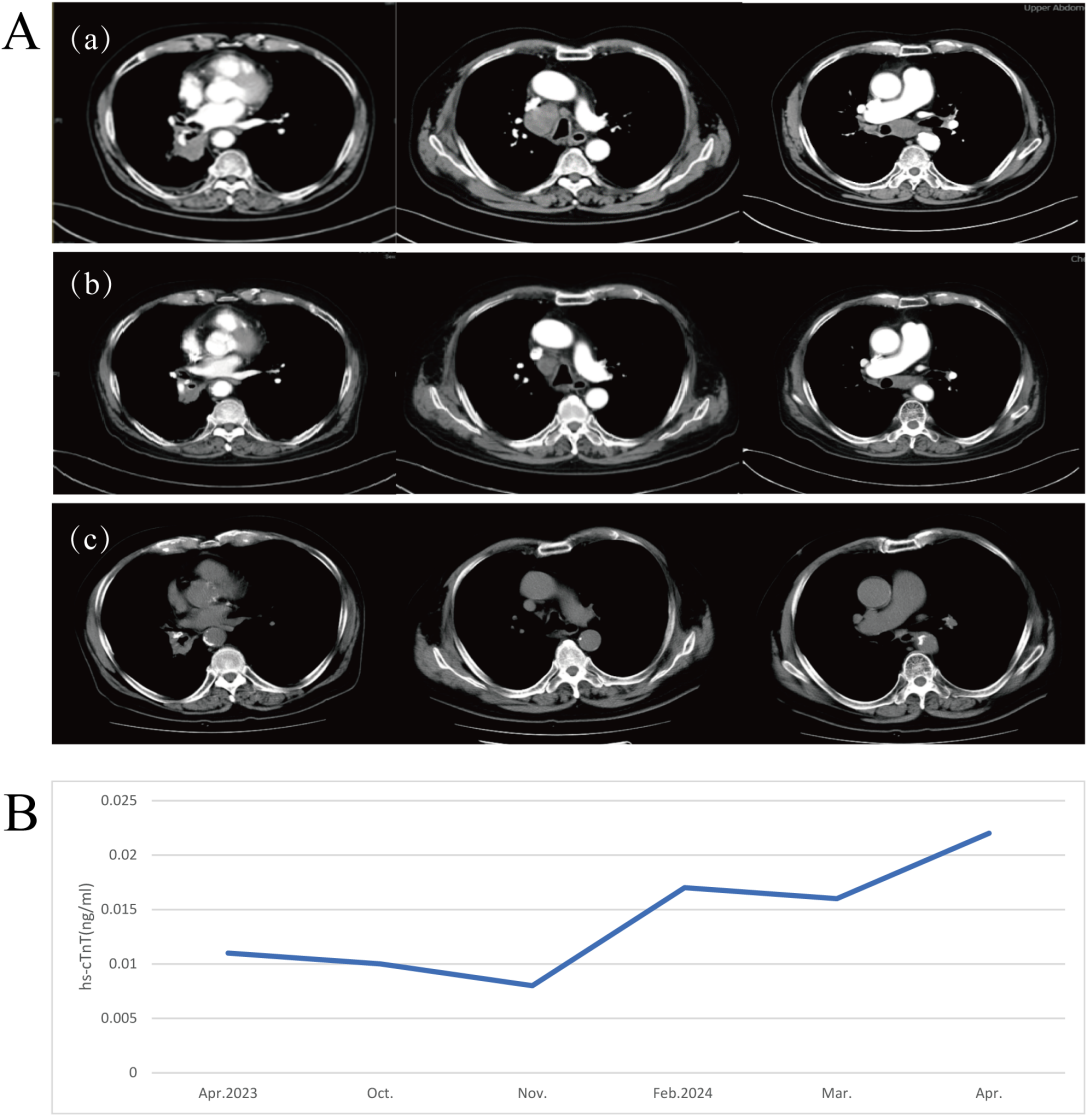
**(A)** Radiographic evaluation of cadonilimab as second-line therapy following disease progression on pembrolizumab. (a) Chest CT performed on October 16, 2023, prior to initiating cadonilimab. (b) Chest CT conducted on December 11, 2023, after three cycles of cadonilimab. (c) Chest CT obtained on March 6, 2025, following 22 cycles of cadonilimab therapy. **(B)** A transient and mild elevation in serum high-sensitivity cardiac troponin T (hs-cTnT) levels was observed during treatment(reference range: 0–0.014 μg/L).

On October 16, 2023, the patient returned to the clinic due to worsening clinical symptoms. Contrast-enhanced CT imaging identified a newly emerged mass in the right lower lobe measuring 49 mm × 67 mm, accompanied by multiple enlarged lymph nodes in the mediastinum, right hilum, and subcarinal regions (**Figure 3A**, region a). Additionally, serum levels of squamous cell carcinoma antigen (SCCA) were elevated, and the therapeutic response was classified as PD. The medical team conducted comprehensive discussions with the patient and his family, clearly outlining the potential benefits and associated risks of available treatment options. Consequently, on October 18, 2023, the patient initiated second-line immunotherapy with cadonilimab (375 mg, 6 mg/kg), administered every three weeks. After completing three treatment cycles, radiological evaluation showed a reduction in the size of the right hilar mass (maximum diameter approximately 45 mm), along with regression of multiple enlarged lymph nodes in both hilar and mediastinal regions (**Figure 3A**, region b). The treatment response was evaluated as PR. The patient reported substantial relief from dyspnea and resumed daily ambulatory activities, comfortably walking more than 500 meters without significant discomfort. His physical condition nearly returned to the best baseline level observed prior to disease progression, and he exhibited a positive and optimistic outlook.

The patient continued regular follow-up visits and maintained stable disease (SD) status until the most recent assessment on March 6, 2025, (**Figure 3A**, region c) achieving a PFS of 17 months. Throughout this period, the patient maintained a satisfactory quality of life.

### 2.4 Immune-related adverse events

**Figure 4:**
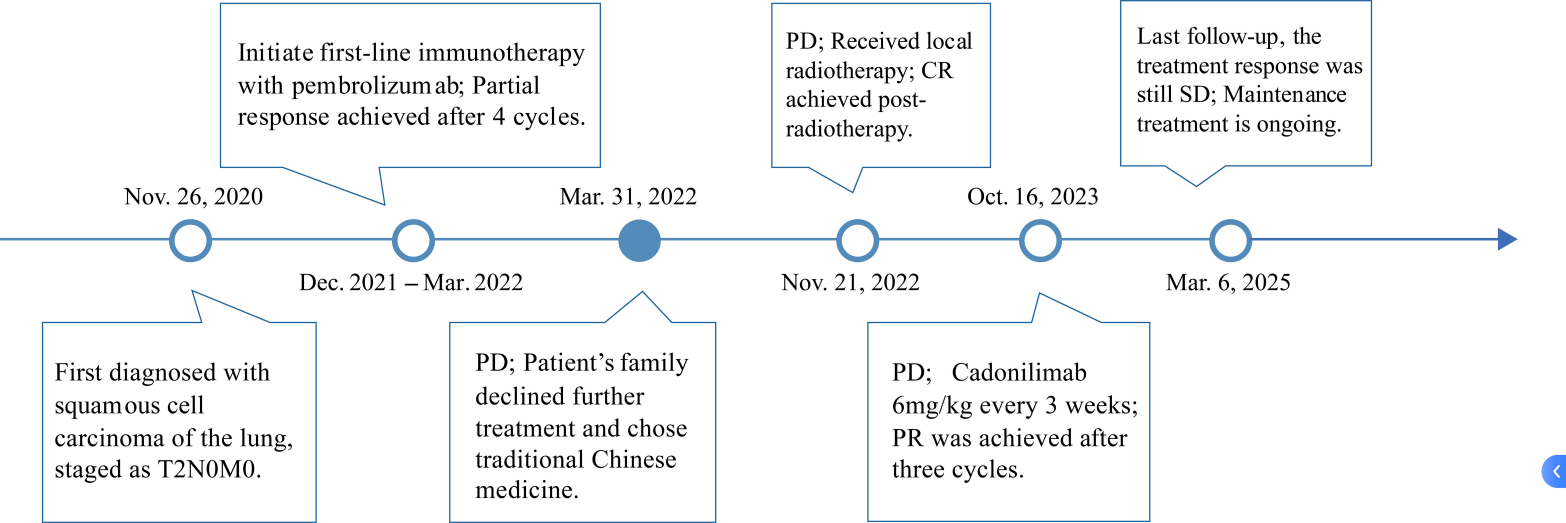
The treatment timeline for the patient. From December 28, 2020, to March 31, 2022, the patient received pembrolizumab therapy. Following disease progression, cadonilimab was initiated on January 22, 2024.

During treatment, the patient exhibited a mild elevation in high-sensitivity cardiac troponin T (hs-cTnT) to 0.22 μg/L, which was classified as Grade 1 according to the Common Terminology Criteria for Adverse Events (CTCAE) version 5.0 (**Figure 3B**). No clinical symptoms such as chest pain or arrhythmia were reported. Electrocardiogram (ECG), echocardiography, and cardiac magnetic resonance imaging (MRI) showed no evidence of myocarditis. Given the patient’s history of coronary artery disease, a referral to the cardiology department was recommended for further evaluation. Following appropriate medical intervention, hs-cTnT levels normalized. Although the transient elevation in hs-cTnT occurred during treatment, the absence of corresponding clinical manifestations and imaging abnormalities did not support a diagnosis of irAEs. Rather, this finding was more likely attributable to underlying cardiovascular pathology. The overall treatment timeline is summarized in **Figure 4**.

## 3 Discussion

This case report describes a patient diagnosed with LUSC who developed acquired resistance following first-line immunotherapy. In the absence of well-established second-line therapeutic options, the patient was treated with cadonilimab monotherapy and achieved a sustained PR, accompanied by favorable treatment tolerance and preservation of quality of life. Although clinical trials evaluating cadonilimab in NSCLC have been initiated, the majority focus on lung adenocarcinoma or heterogeneous patient populations without stratification by histological subtype. Clinical evidence remains limited for LUSC—a distinct subtype characterized by a more complex tumor immune microenvironment and fewer effective therapeutic strategies—particularly in patients who have developed acquired resistance to ICIs. Notably, this patient was elderly and presented with multiple comorbidities, including hypertension, COPD, and coronary artery disease—clinical features that frequently result in exclusion from clinical trials. Despite these risk factors, the patient achieved both radiological disease control and significant clinical improvements in key symptoms such as chest tightness, cough, and fatigue. Furthermore, the patient exhibited enhanced emotional well-being and improved functional capacity in daily activities, reflecting a clinically meaningful enhancement in overall quality of life. This case provides a novel perspective on the management of immunotherapy-resistant LUSC and offers valuable insights into the treatment of high-risk, complex patients in real-world clinical practice.

For patients with early-stage NSCLC, surgical resection remains the established curative treatment. However, for those deemed inoperable due to poor general condition or significant comorbidities, stereotactic body radiotherapy (SBRT), also referred to as stereotactic ablative radiotherapy (SABR), has emerged as a preferred alternative, offering excellent local control and a favorable toxicity profile (6). Accumulating evidence indicates that the I-SABR regimen, which combines PD-1 blockade with SABR, can significantly improve 4-year event-free survival (EFS) rates (7). In this case, as the patient declined radiotherapy, we referenced key clinical trials, including KEYNOTE-001, KEYNOTE-024, and KEYNOTE-042, to inform our treatment decision. Based on these data, pembrolizumab monotherapy was selected, given its demonstrated superior efficacy and more favorable safety profile compared to chemotherapy in patients with high PD-L1 expression ([TPS] ≥ 50%) (8, 9).

However, the selection of an optimal subsequent-line regimen for NSCLC remains a significant clinical challenge. Docetaxel is frequently utilized as a second-line therapeutic option; however, its efficacy is modest and is accompanied by considerable toxicity. For example, a pivotal phase III trial reported an objective response rate(ORR)of only 6.7%, with a median progression-free survival (mPFS) of 10.6 weeks (10). In patients experiencing oligoprogressive disease following first-line immunotherapy, localized treatment strategies centered on radiotherapy may provide effective control of progressive lesions. The continuation of ICIs after radiotherapy may reinvigorate antitumor immune responses in select patients, thereby offering potential clinical benefit (11).

Within this clinical context, ICI rechallenge has emerged as a promising area of research in oncology. A retrospective study by Xu et al. demonstrated that continuation of immunotherapy beyond initial disease progression—particularly in cases of oligoprogression—is both feasible and associated with clinical efficacy (12). Moreover, key clinical trials such as CheckMate 017, CheckMate 057, and OAK have established the efficacy of conventional ICIs (e.g., nivolumab, atezolizumab) as second-line therapies, showing improved overall survival and a more favorable safety profile compared to chemotherapy (13, 14). Additionally, incorporating chemotherapy before ICI rechallenge may lead to improved therapeutic outcomes (15, 16). Combination regimens involving PD-1/PD-L1 inhibitors and CTLA-4 inhibitors have demonstrated superior clinical efficacy compared to monotherapy in multiple randomized trials—including POSEIDON, CheckMate 227, and CheckMate 032—regardless of tumor histology or PD-L1 expression levels (17–19). Nevertheless, the increased risk of irAEs associated with this approach limits its widespread clinical adoption (20).

Notably, in NSCLC patients who develop acquired resistance to immunotherapy, the tumor microenvironment (TME) often undergoes a transition toward an immunosuppressive state. This transformation is characterized by reduced infiltration of effector CD8^+^ T cells, impaired antigen presentation, increased populations of immunosuppressive cells such as regulatory T cells (Tregs) and myeloid-derived suppressor cells (MDSCs), decreased levels of proinflammatory cytokines like IFN-γ, and upregulation of alternative coinhibitory pathways including CTLA-4, LAG-3, and TIM-3. Collectively, these mechanisms constrain antitumor immune responses, leading to a “cold tumor” phenotype and diminishing the sustained efficacy of conventional PD-1/PD-L1 inhibitors (21, 22). In this case, although first-line immunotherapy initially conferred clinical benefit, the subsequent emergence of acquired resistance indicates a dynamic shift in the TME from a “hot” to a “cold” state. Furthermore, LUSC is inherently more immunologically heterogeneous than adenocarcinoma, with greater enrichment of Tregs and aberrant activation of inflammation-related signaling pathways (23). Cadonilimab, a bispecific tetravalent antibody, simultaneously binds to PD-1 and CTLA-4 with high affinity—substantially stronger than conventional monoclonal antibodies. This dual-targeting mechanism enhances initial T-cell activation and tumor cytotoxicity, suppresses Treg activity, and promotes immune cell infiltration into the tumor, thereby reversing the immunosuppressive TME through multiple pathways. These properties render Cadonilimab particularly suitable for patients with high Treg infiltration and a background of immunotherapy resistance. Importantly, its engineered Fc-silent (Fc null) structure minimizes immune-related toxicity without compromising antitumor efficacy (5, 24, 25). Clinical studies have demonstrated that Cadonilimab exhibits a favorable safety profile, particularly showing a marked reduction in high-grade irAEs, such as colitis and hypophysitis, compared to conventional CTLA-4 inhibitors (4). Moreover, preclinical and clinical evidence suggests that combining anti-angiogenic therapy can normalize aberrant tumor vasculature, modulate the TME, enhance infiltration of immune effector cells, and ultimately improve the efficacy of ICIs (26, 27). However, in this case, anti-angiogenic agents were not administered due to the patient’s poorly controlled hypertension.

In the field of NSCLC, several clinical studies have yielded promising results, although research remains in early stages. The preliminary findings of the AK104-IIT-018 study, presented at the Asian Lung Cancer Conference (ALCC), showed that the combination of Cadonilimab with anlotinib and docetaxel achieved encouraging efficacy in patients with immunotherapy-resistant NSCLC. As of May 31, 2024, 46 patients had been enrolled and treated with the combination regimen, with a median follow-up duration of 2.8 months. Early analysis revealed notable antitumor activity in this challenging population, with a 6-month PFS rate of 56.9%, a mPFS of 6.5 months, an ORR of 30.3%, and a disease control rate (DCR) of 94.0%. Another multicenter retrospective study included 41 patients with advanced NSCLC who had developed resistance to PD-1/PD-L1 inhibitors. Over half received fourth-line or later treatment regimens based on Cadonilimab. The results showed an ORR of 4.3%, a DCR of 39.1%, and a mPFS of 108 days; treatment-related adverse events were generally manageable (28). Furthermore, the COMPASSION-01 study demonstrated that Cadonilimab retained antitumor activity even in patients who had received multiple prior lines of therapy or exhibited resistance to ICIs. Notably, more than half of the 18 patients with prior immunotherapy resistance achieved either partial response PR or SD (29). These findings suggest that Cadonilimab holds promise in overcoming the therapeutic limitations associated with resistance to first-line immunotherapy, potentially expanding clinical benefit for patients with advanced NSCLC. Additionally, differentiating between primary and acquired immunotherapy resistance is critical for guiding subsequent treatment strategies. The AK104–202 study stratified patients according to the type of immunotherapy resistance and found that those with acquired resistance had significantly longer overall survival compared to patients with primary resistance (13.16 months vs. 4.93 months), indicating that rechallenging immunotherapy may still confer a survival advantage in selected patients (30).

In this case, the patient developed acquired resistance following first-line immunotherapy and subsequently achieved a partial response with Cadonilimab monotherapy in the absence of additional therapeutic agents. The PFS exceeded 17 months, which is substantially longer than the median PFS reported in the COMPASSION-01 and AK104-IIT-018 studies. This favorable clinical outcome is consistent with findings from the AK104–202 trial, which indicated that patients with acquired resistance are more likely to derive clinical benefit from immunotherapy rechallenge compared to those with primary resistance. Furthermore, this patient represents a subgroup often excluded from clinical trials—elderly individuals with multiple comorbidities and a diagnosis of lung squamous cell carcinoma—thereby highlighting the real-world applicability and clinical relevance of this case. Therefore, this case not only supports existing research findings regarding efficacy but also contributes valuable insights into histological subtype, patient characteristics, and treatment strategies.

With regard to biomarkers, although PD-L1 remains the primary predictive biomarker for immunotherapy, its predictive performance exhibits considerable heterogeneity across studies, and the correlation between PD-L1 expression levels and immunotherapy response remains inconsistent (31). A real-world study in patients with recurrent or metastatic cervical cancer found no significant association between Cadonilimab efficacy and PD-L1 expression. Notably, patients with negative PD-L1 expression achieved an ORR comparable to or even higher than that observed with conventional PD-1 inhibitors (32). This discrepancy may be attributed to several factors, including variability in detection methodologies, spatial and temporal heterogeneity of PD-L1 expression, and subjectivity in interpretation, all of which limit the utility of PD-L1 as a standalone predictive biomarker. In contrast, tumor mutational status, particularly mutations in driver genes such as EGFR, KRAS, and STK11, has recently emerged as a more stable predictor of immunotherapy response. These genetic alterations influence the tumor microenvironment and immune cell infiltration through key signaling pathways such as IL-6/JAK/STAT3, thereby significantly affecting immunotherapy sensitivity (33, 34). In this case, the patient had lung squamous cell carcinoma and was negative for known driver gene mutations. In the absence of clear immunophenotypic guidance, the driver gene-negative status may represent a potential subgroup that could benefit from Cadonilimab therapy.

Although this case provides encouraging evidence supporting the use of Cadonilimab monotherapy as a rechallenge strategy, several limitations must be acknowledged. First, invasive procedures such as re-biopsy or immunophenotyping were not feasible due to the patient’s advanced age and comorbidities, which posed significant clinical and ethical concerns. As a result, we were unable to conduct in-depth mechanistic analyses of tumor microenvironmental changes, immune cell infiltration, PD-L1 expression dynamics, and molecular features associated with acquired resistance. Second, the findings from this single-case report cannot be generalized to a broader patient population, and the patient’s clinical complexity further limits the applicability of these observations. Third, LUSC itself presents unique challenges due to its complex tumor immune microenvironment and limited available research on immune resistance mechanisms, which inherently limits opportunities for comprehensive mechanistic exploration in single-case studies. The efficacy and safety profile of Cadonilimab across different patterns of immunotherapy resistance remains to be fully elucidated. Therefore, larger-scale, high-quality clinical studies are warranted to confirm the true efficacy and safety of Cadonilimab in patients with immunotherapy-resistant lung squamous cell carcinoma.

## 4 Conclusion

In conclusion, cadonilimab, a bispecific monoclonal antibody targeting both PD-1 and CTLA-4, demonstrates promising potential not only in overcoming immune resistance but also in enhancing quality of life—particularly among elderly patients for whom standard treatment options are often suboptimal or contraindicated. Although further clinical investigation is required to fully validate its impact on survival outcomes, cadonilimab represents a valuable therapeutic option by improving symptom management and functional status in this high-risk and underserved patient population. Furthermore, future research should prioritize dose optimization, the refinement of patient selection criteria, and the identification of predictive biomarkers to further advance the implementation of precision immunotherapy in real-world clinical practice.

## Data Availability

The original contributions presented in the study are included in the article/**Supplementary Material**, Further inquiries can be directed to the corresponding author.
