# Supplementary material for: Cadonilimab as second-line therapy in immunotherapy-resistant squamous NSCLC: a case report and review
Source: Front Immunol. 2025 Jul 24;16:1627147. doi: 10.3389/fimmu.2025.1627147 (PMC12328319; doi:10.3389/fimmu.2025.1627147)
Supplement: Supplementary file 1 [file DataSheet1.pdf]

CARE Checklist of information to include when writing a case report

CARE case report guidelines

(cc) BY-NC-ND

Topic	Item	Checklist item description	Reported on Line
**Title**	1	The diagnosis or intervention of primary focus followed by the words "case report"	1
**Key Words**	2	2 to 5 key words that identify diagnoses or interventions in this case report, including "case report"	8-9
**Abstract (no references)**	3a	Introduction: What is unique about this case and what does it add to the scientific literature?	
	3b	Main symptoms and/or important clinical findings	
	3c	The main diagnoses, therapeutic interventions, and outcomes	15-18
	3d	Conclusion—What is the main "take-away" lesson(s) from this case?	18
**Introduction**	4	One or two paragraphs summarizing why this case is unique (**may include references**)	20-42
**Patient Information**	5a	De-identified patient specific information.	44-49
	5b	Primary concerns and symptoms of the patient.	44-45
	5c	Medical, family, and psycho-social history including relevant genetic information	
	5d	Relevant past interventions with outcomes	
**Clinical Findings**	6	Describe significant physical examination (PE) and important clinical findings.	
**Timeline**	7	Historical and current information from this episode of care organized as a timeline	
**Diagnostic Assessment**	8a	Diagnostic testing (such as PE, laboratory testing, imaging, surveys).	50-59
	8b	Diagnostic challenges (such as access to testing, financial, or cultural)	
	8c	Diagnosis (including other diagnoses considered)	57
	8d	Prognosis (such as staging in oncology) where applicable	
**Therapeutic Intervention**	9a	Types of therapeutic intervention (such as pharmacologic, surgical, preventive, self-care)	
	9b	Administration of therapeutic intervention (such as dosage, strength, duration)	63-64, 72-73, 80-81
	9c	Changes in therapeutic intervention (with rationale)	67-69, 75-79
**Follow-up and Outcomes**	10a	Clinician and patient-assessed outcomes (if available)	83-85
	10b	Important follow-up diagnostic and other test results	
	10c	Intervention adherence and tolerability (How was this assessed?)	
	10d	Adverse and unanticipated events	85-87
**Discussion**	11a	A scientific discussion of the strengths AND limitations associated with this case report	88-184
	11b	Discussion of the relevant medical literature **with references**.	222-289
	11c	The scientific rationale for any conclusions (including assessment of possible causes)	
	11d	The primary "take-away" lessons of this case report (without references) in a one paragraph conclusion	185-194
**Patient Perspective**	12	The patient should share their perspective in one to two paragraphs on the treatment(s) they received	
**Informed Consent**	13	Did the patient give informed consent? Please provide if requested	Yes ☑ No ☐
